# A pictorial essay on cross-sectional imaging findings of pathologies in the second (D2) segment of the duodenum in adults

**DOI:** 10.1007/s00261-025-04846-7

**Published:** 2025-02-23

**Authors:** Isil Basara Akin, Muhammed Enes Oguzturk, Bengisu Kandemir, Nihal Deniz Mentes, Canan Altay

**Affiliations:** Department of Radiology, School of Medicine, Dokuz Eylül University, Izmir, Turkey

**Keywords:** Computed tomography, Duodenum, Magnetic resonance imaging, Neoplastic pathologies, Non-neoplastic pathologies

## Abstract

The duodenum, the initial segment of the small intestine, is divided into four parts: the superior (D1), descending (second) (D2), horizontal (D3), and ascending (D4) segments. Despite its short length, the descending part (D2 segment) holds clinical significance due to its anatomical proximity to structures such as the gallbladder, right kidney, colon, and pancreas. This anatomical localization and contiguity give rise to various pathologies, including congenital, inflammatory, infectious, neoplastic, vascular, and traumatic conditions. Cross-sectional imaging modalities play a pivotal role in evaluating pathologies of the second (D2) segment of the duodenum. This article aims to provide a comprehensive overview of these pathologies and delineate their imaging characteristics.

## Introduction

The duodenum, the proximal segment of the small intestine, maintains close anatomical relationships with retroperitoneal structures such as the pancreas, ascending colon, spine, and aorta, as well as intraperitoneal structures like the stomach, liver, and gallbladder. Duodenal pathologies are frequently underdiagnosed both clinically and radiologically [1].

Structurally, the duodenum forms a C-shaped curve, spanning the L1–L3 vertebral levels on the right side of the vertebral column [2]. It extends from the duodenal bulb to the ligament of Treitz and consists of four distinct segments: superior, descending, horizontal, and ascending. Except for the superior segment, which is intraperitoneal, the duodenum is primarily retroperitoneal.

**Fig. 1:**
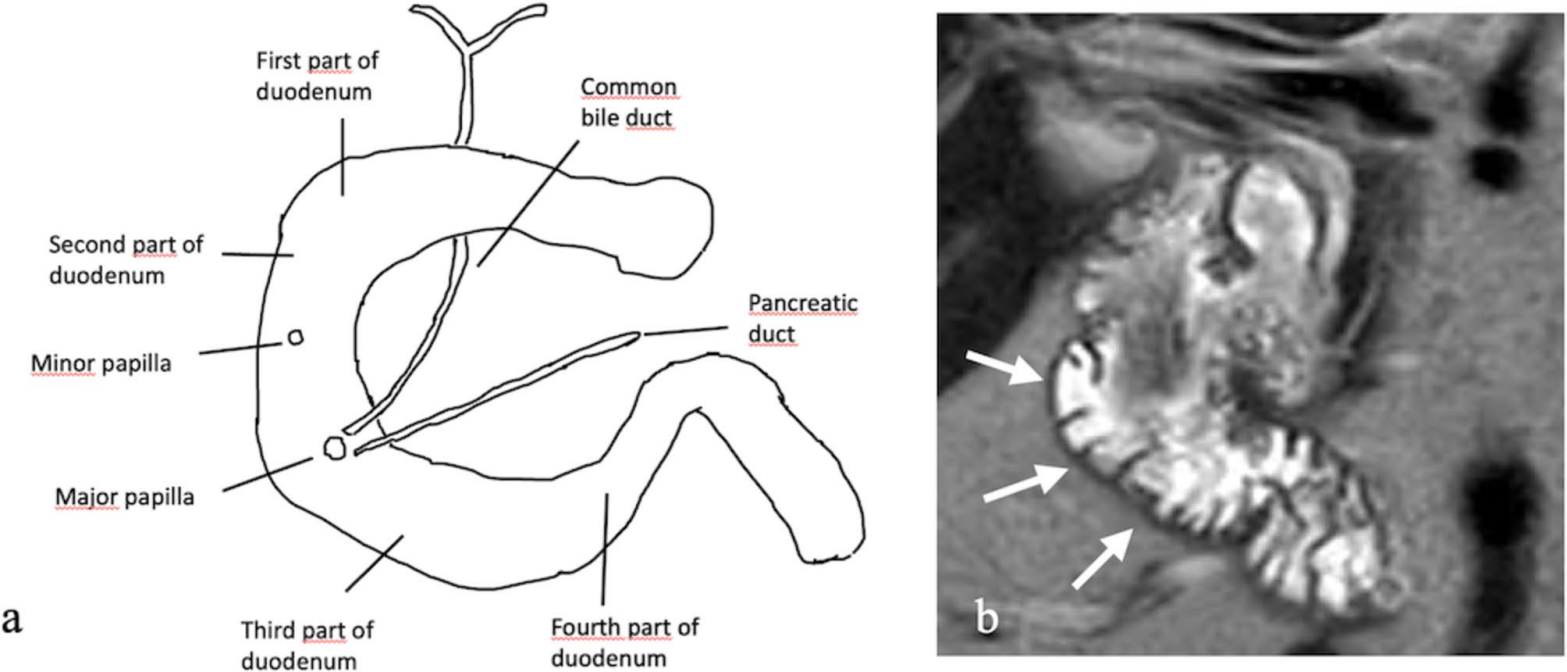
a. Anatomical illustration of the duodenum. **b**. Coronal T2-weighted MRI image demonstrating the second (D2) segment of the duodenum (arrows)

The descending, or second (D2), segment is located within the transverse mesocolon anteriorly and bordered posteriorly by the right adrenal gland, kidney, and ureter. Superiorly, it is adjacent to the gallbladder and liver, while inferiorly, it is bordered by jejunal loops. Laterally, it is flanked by the ascending colon, hepatic flexure, and right kidney, while medially, it is closely related to the pancreatic head (Fig. 1). This segment also houses critical anatomical landmarks, including the Ampulla of Vater and the minor papilla [3].

Pathologies affecting the D2 segment of the duodenum can arise from intrinsic factors, including congenital, inflammatory, infectious, neoplastic, vascular, and traumatic etiologies, as well as extrinsic factors from adjacent pathological processes [1, 4]. The D2 segment is often implicated in various clinical conditions, making accurate and timely diagnosis essential for appropriate treatment strategies [4].

Cross-sectional imaging modalities, particularly computed tomography (CT), play a crucial role in evaluating the duodenal wall, intraluminal contents, periduodenal space, and vascular structures. CT is especially valuable in emergency settings, while magnetic resonance imaging (MRI) is frequently utilized for further characterization of pathologies. These imaging techniques aid in differential diagnosis and guide optimal treatment approaches [4]. Table 1 provides a summary of neoplastic and non-neoplastic entities along with their imaging features.

**Table 1:** Neoplastic and non-neoplastic pathologies and imaging findings of second—D2 segment of the duodenum

Pathologies in the second-D2 segment of the duodenum		Imaging findings
Congenital Pathologies	Duodenal duplication cyst	∙ Fluid-filled structures with smooth walls, typically situated near the duodenum but distinct from the pancreas and biliary system
	Ectopic pancreas	∙ Intramural or endoluminal mass with homogeneous contrast enhancement ∙ MRI findings include a high-intensity signal on T1-weighted images
	Annular pancreas	∙ CT imaging reveals pancreatic tissue encircling the second—(D2) segment of the duodenum
Non-Neoplastic Pathologies	Duodenal diverticulum and diverticulitis	∙ Protrusions from duodenal wall, typically containing an air-fluid level ∙ Non-specific inflammatory changes in diverticulitis
	Inflammatory and infectious conditions	∙ Imaging findings are mostly non-specific and differ according to different diagnoses
	Paraduodenal pancreatitis	∙ Fat stranding in the pancreaticoduodenal groove and cystic changes in the second—(D2) segment of the duodenum
	Duodenal perforation	∙ CT is the gold standard in the diagnosis ∙ Focal wall defects, extraluminal free air
	Bouveret’s Syndrome	∙ CT imaging shows the classic diagnostic triad—Rigler’s triad
	Duodenal hematoma	∙ High-density in subacute hematoma. Increased lesion density in late-stage Active bleeding sites may be seen on contrast-enhanced CT ∙ MRI typically shows a hyperintense lesion on T1-weighted images
Neoplastic Pathologies	Duodenal lipoma	∙ Lesions with fat density on CT images ∙ High signal intensity on T1- and T2-weighted on MRI images
	Duodenal polyp	∙ Well-defined soft tissue lesions, either sessile or pedunculated ∙ Mild and homogeneous contrast enhancement
	Leiomyoma	∙ Well-defined solid masses ∙ Calcification or ulceration may exhibit ∙ MRI findings typically demonstrate homogeneous signal intensity
	Gastrointestinal stromal tumors	∙ On CT well-demarcated hypervascular lesions with homogeneous contrast enhancement ∙ Necrosis, cystic changes, or central calcifications may be seen in large tumors ∙ Low T1 and high T2 signal intensity on MRI
	Neuroendocrine tumors	∙ Early arterial-phase enhancement. MRI ∙ Hyperintense cystic components on T2 MRI images ∙ Periduodenal desmoplastic reactions and enhancing lymph nodes,
	Duodenal adenocarcinomas	∙ Asymmetric or concentric wall thickenings or mass lesions with polypoid growth patterns ∙ Necrosis, heterogeneous late contrast enhancement ∙ Double-duct sign is frequently observed in periampullary adenocarcinomas ∙ Low T2 signal intensity relative to intraluminal contents on MRI ∙ Diffusion restriction
	Duodenal lymphomas	∙ Aneurysmal dilatation is a hallmark feature ∙ Splenomegaly, mesenteric lymphadenopathy
	Metastases	∙ Imaging findings are nonspecific

*CT* computed tomography, *MRI* magnetic resonance imaging

This review aims to elucidate the pathologies involving the D2 segment of the duodenum, encompassing both neoplastic and non-neoplastic conditions, and to highlight the imaging findings that facilitate accurate diagnosis and effective clinical management.

## Congenital pathologies

### Duodenal duplication cyst

Duodenal duplication cysts are rare congenital anomalies, accounting for 5–7% of gastrointestinal tract cysts. These cysts most commonly occur in the D1 and D2 segments of the duodenum, originating from the muscular layer of the medial wall [1]. Larger cysts can cause obstruction, pancreatitis, nausea, vomiting, abdominal pain, or obstructive jaundice. In rare cases, malignant transformation has been reported [5].

**Fig. 2:**
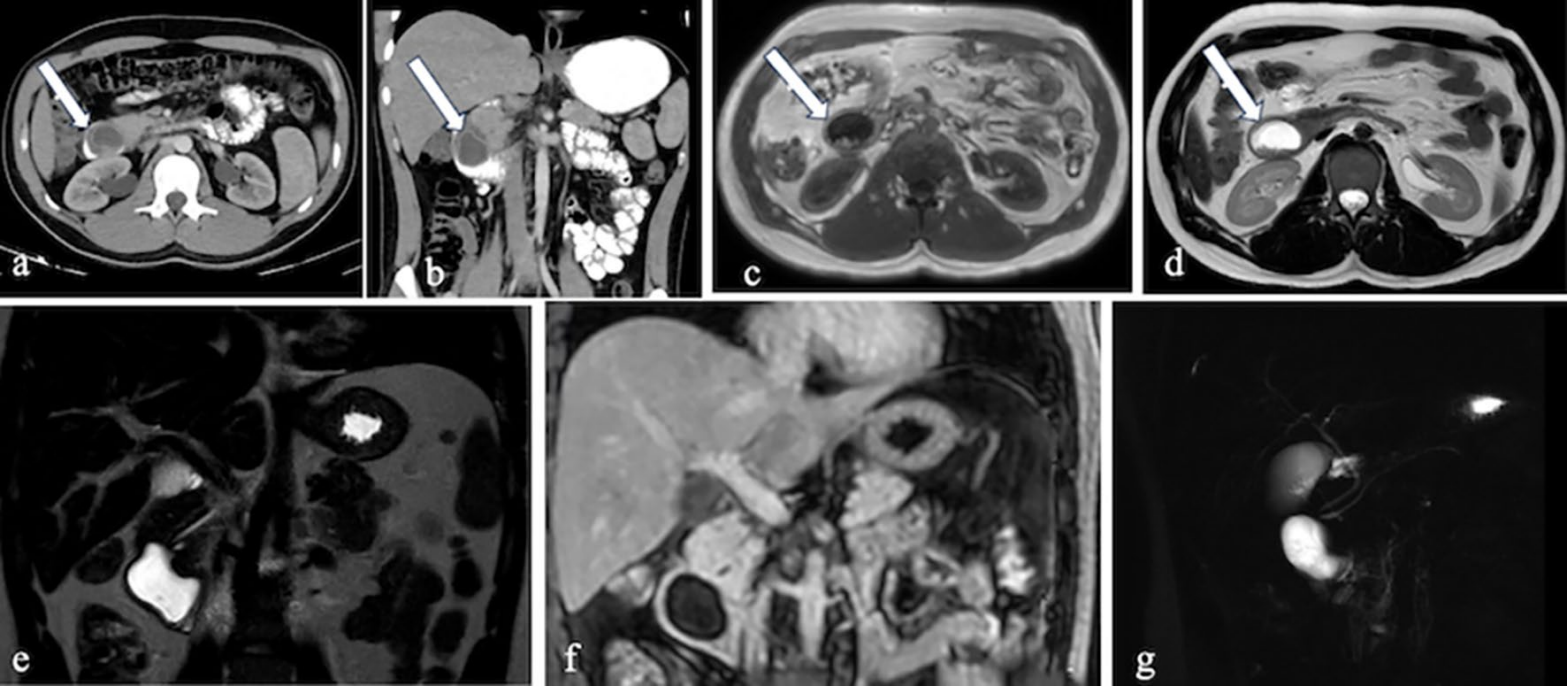
a. Axial and **b**. Coronal CT images with positive oral and intravenous (IV) contrast administration, **c–g** MRI images of a 25-year-old male patient with a duodenal duplication cyst, including axial T1-weighted (**c**), axial T2-weighted (**d**), coronal T2-weighted (**e**), coronal post-contrast fat-saturated T1-weighted (**f**), and MRCP (**g**) sequences. The cystic lesion exhibits no contrast enhancement. Multiple millimetric hypointense signals, consistent with millimetric stones, are observed on MRI. MRCP reveals no connection with the pancreaticobiliary system (arrows).

On CT, these cysts appear as fluid-filled structures with smooth walls, typically located near the duodenum but distinct from the pancreas and biliary system. MRI findings show cystic lesions that are hypointense on T1-weighted images and hyperintense on T2-weighted images. Magnetic resonance cholangiopancreatography (MRCP) and endoscopic retrograde cholangiopancreatography (ERCP) are instrumental in evaluating their connection to the pancreaticobiliary system [6] (Fig. 2).

### Ectopic pancreas

Ectopic pancreas, or heterotopic pancreas, is a congenital anomaly characterized by pancreatic tissue located outside its normal anatomical site, without vascular or ductal continuity with the main pancreas. Its prevalence ranges from 0.5% to 13% in autopsy series and 0.2%–0.9% in upper abdominal surgeries, with the duodenum being the most frequent location [7].

**Fig. 3:**
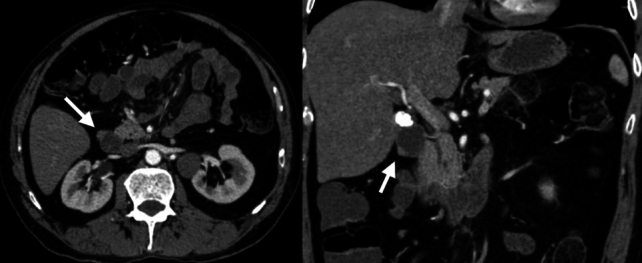
Axial and coronal negative oral and IV contrast-enhanced CT images of a 57-year-old male patient. A nodular lesion with density and enhancement characteristics similar to pancreatic parenchyma is observed, consistent with ectopic pancreatic tissue (arrows)

On CT, ectopic pancreas appears as an intramural or endoluminal mass with homogeneous contrast enhancement, displaying a similar density to normal pancreatic tissue. MRI findings include high signal intensity on T1-weighted images and homogeneous enhancement after contrast administration, resembling the signal characteristics of normal pancreas [7] (Fig. 3).

### Annular pancreas

Annular pancreas is a rare congenital anomaly in which a ring of pancreatic tissue encircles the second (D2) segment of the duodenum, either partially or completely. This condition commonly presents in infancy with gastric outlet obstruction symptoms but may be incidentally discovered in adults during evaluations for pancreatitis or peptic ulcer disease. The estimated prevalence of annular pancreas is 5–15 per 100,000 autopsies [8].

Developmentally, functionally, and anatomically, the duodenum is closely associated with other structures such as the celiac trunk and pancreas. Congenital anomalies affecting these structures are rare, and annular pancreas is one such anomaly. Duodenal atresia and stenosis may also be associated with annular pancreas [9, 10]. "Head-over-heels" positioning is another rare congenital anomaly, characterized by a difference in the extension of the D1 and D2 segments of the duodenum. Additionally, duplication and malrotation are other known duodenal variations [9].

**Fig. 4:**
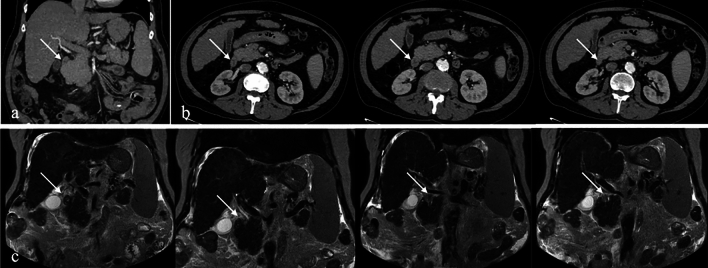
IV contrast-enhanced **a**. Coronal and **b**. Axial CT images. **c**. Coronal T2-weighted MRI image of a 58-year-old male patient with chronic liver disease. The images demonstrate annular pancreatic tissue encircling the second (D2) segment of the duodenum (arrows), consistent with annular pancreas

CT imaging reveals pancreatic tissue encircling the D2 segment of the duodenum. MRI findings are consistent with CT, providing additional soft tissue contrast to evaluate ductal and parenchymal involvement [8] (Fig. 4).

### Non-neoplastic pathologies

#### Duodenal diverticulum and diverticulitis

Duodenal diverticula most commonly arise from the second (D2) segment of the duodenum, particularly in the juxtapapillary region. They are classified as either true or false diverticula. True diverticula are congenital anomalies involving all layers of the duodenal wall, whereas false diverticula are acquired, resulting from mucosal, muscularis mucosa, and submucosal herniation through weak points in the wall, often near vascular structures [11]. The prevalence of duodenal diverticula ranges from 10 to 20% [11].

**Fig. 5:**
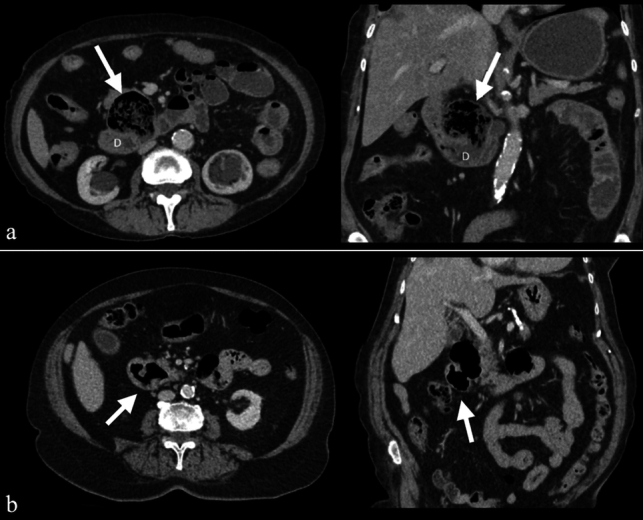
Axial and coronal IV contrast-enhanced CT images from two different patients (47-year-old male and 53-year-old female) illustrating duodenal diverticula in the second (D2) segment. The diverticulum in the first patient contains intestinal contents (**a**), while in the second patient, it contains only air (**b**) (arrows)

**Fig. 6:**
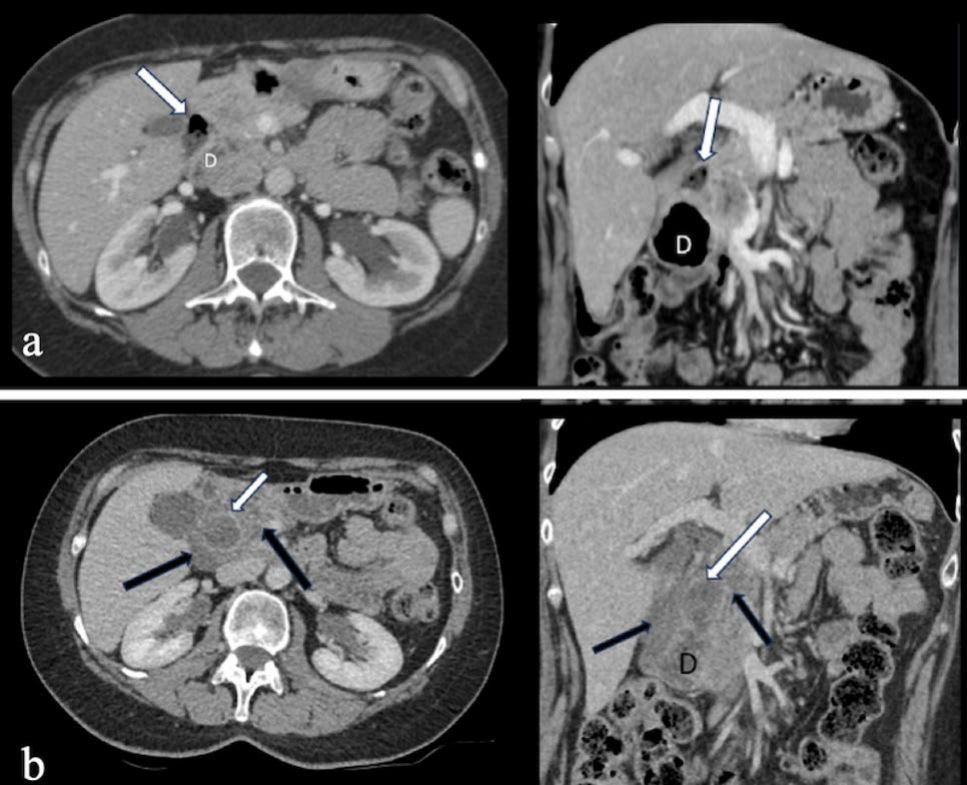
Axial and coronal IV contrast-enhanced CT images of a 61-year-old female patient. **a**. CT images obtained five years prior show a duodenal diverticulum (white arrows). **b**. Recent CT images reveal significant duodenal wall thickening, hyperenhancement, periduodenal heterogeneity, and fluid collections (black arrows), findings consistent with duodenal diverticulitis

Duodenal diverticula are clinically significant as they can contribute to pancreaticobiliary complications [11]. On CT imaging, duodenal diverticula appear as protrusions from the duodenal wall, typically containing an air-fluid level, but sometimes appearing with only air, fluid, or intestinal contents (Fig. 5). Although generally asymptomatic, complications such as inflammation, bleeding, ischemia, and perforation may occur in rare cases [12]. In complicated cases, CT imaging may reveal findings that mimic other pathologies, including pancreatic head tumors and biliary tract diseases [12] (Fig. 6).

#### Inflammatory and infectious conditions

Peptic ulcer disease is a common condition, affecting 5%–10% of the global population. The primary etiological factor is Helicobacter pylori infection, followed by nonsteroidal anti-inflammatory drug (NSAID) use. The second (D2) segment of the duodenum, particularly the bulb, is the most frequent site of ulceration [13].

Pancreatitis is a significant inflammatory cause of duodenitis, affecting the duodenum either directly through pancreatic enzyme damage or indirectly via pancreatic enlargement, peripancreatic inflammation, and fluid collections. Acute cholecystitis can also result in secondary duodenitis due to inflammation in adjacent structures [4].

Crohn’s disease, though primarily affecting the ileum and colon, involves the duodenum in approximately 4%–5% of cases. Classic imaging findings include strictures and ulcers. Advanced stages may present with fistulous connections to adjacent bowel segments [14].

Sarcoidosis rarely affects the gastrointestinal tract, with the stomach being the most commonly involved site. Duodenal involvement is infrequent but may manifest as mucosal granulomas, obstructive changes, malabsorption, and gastrointestinal bleeding [15].

Primary duodenal tuberculosis is a rare infectious entity, accounting for 1%–6% of gastrointestinal tuberculosis cases. Due to its nonspecific clinical and imaging features, diagnosis is often challenging [16].

**Fig. 7:**
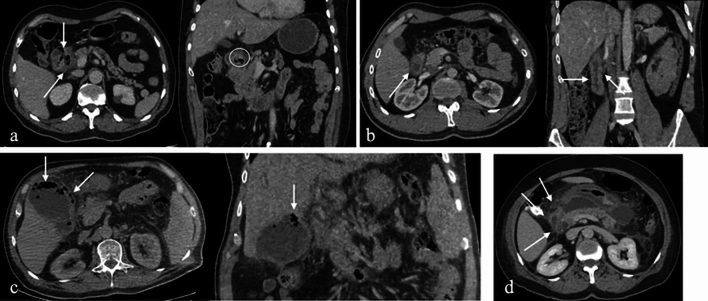
a. Axial and coronal IV contrast-enhanced CT images of a 43-year-old male patient with peptic ulcer disease, showing discontinuity in the duodenal wall, wall thickening, enhancement, and paraduodenal heterogeneity. **b**. CT images of a 38-year-old female patient displaying duodenal wall thickening and enhancement, consistent with non-specific duodenitis. **c**. CT images of a 69-year-old female patient with emphysematous cholecystitis-induced duodenitis, demonstrating air densities within the gallbladder wall. **d**. CT images of a patient with duodenitis secondary to pancreatitis, revealing non-specific duodenal wall thickening

**Fig. 8:**
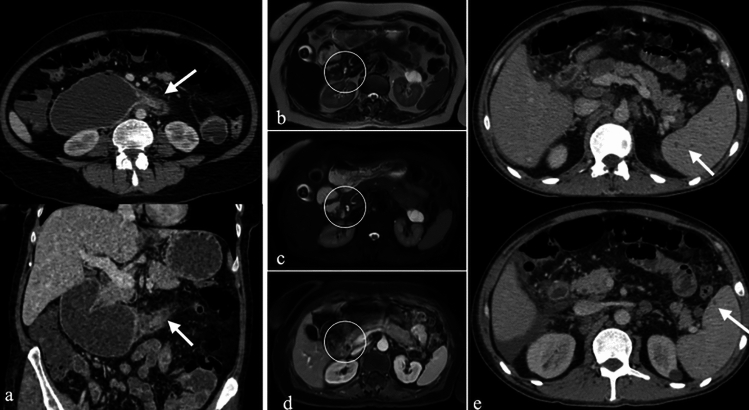
a. Axial and coronal IV contrast-enhanced CT images of a 42-year-old female patient with Crohn’s disease, showing short-segment stenosis, wall thickening, and submucosal fat deposition at the third (D3) segment of the duodenum (arrows), as well as aneurysmal dilatation of the second (D2) segment. MRI images **b**. T2-weighted, **c**. fat-saturated T2-weighted, and **d**. post-contrast fat-saturated T1-weighted) of a 51-year-old female patient with sarcoidosis, showing mucosal thickening and luminal narrowing (circles). **e**. IV contrast-enhanced CT images of a 68-year-old male patient with duodenal tuberculosis, revealing wall thickening, enhancement, and paraduodenal heterogeneity (arrows), along with intra-abdominal free fluid, splenomegaly, and multiple splenic tuberculous lesions

CT imaging is critical in identifying these inflammatory and infectious conditions. Peptic ulcers typically present as segmental mural enhancement with discontinuity, whereas duodenal wall thickening near the gallbladder is characteristic of cholecystitis-induced duodenitis. Findings such as fistulas, short segment stenoses, and mucosal enhancement are indicative of Crohn’s disease. Sarcoidosis and tuberculosis may mimic Crohn’s disease but can be distinguished by specific imaging patterns, such as aneurysmal dilatation or granulomatous lesions [14, 15] (Figs. 7 and 8).

#### Paraduodenal pancreatitis

Paraduodenal pancreatitis, also known as groove pancreatitis, is a rare form of focal chronic pancreatitis affecting the pancreaticoduodenal groove—a potential space between the pancreatic head, common bile duct, and the second (D2) segment of the duodenum [17]. This condition is characterized by an inflammatory mass-forming process within this space, leading to solid wall thickening of the duodenum and, in some cases, cystic changes at the center of the groove area [18].

Paraduodenal pancreatitis is classified into two types: solid and cystic, with the cystic type being the more common form [19, 20]. Inflammatory changes may also extend to the entire pancreatic tissue due to obstruction of the pancreatic duct, leading to obstructive pancreatitis [18]. The most frequent clinical symptoms include abdominal pain, duodenal and common bile duct obstruction resulting in jaundice, weight loss, and vomiting. In advanced cases, pancreatic insufficiency symptoms such as steatorrhea and diabetes may occur [18].

The prevalence of paraduodenal pancreatitis in the literature ranges from 3.5% to 6% [18]. It predominantly affects middle-aged male patients, particularly heavy smokers and alcohol consumers. Due to its overlapping symptoms with pancreatic cancer, distinguishing between the solid type of paraduodenal pancreatitis and malignancy can be challenging [18].

**Fig. 9:**
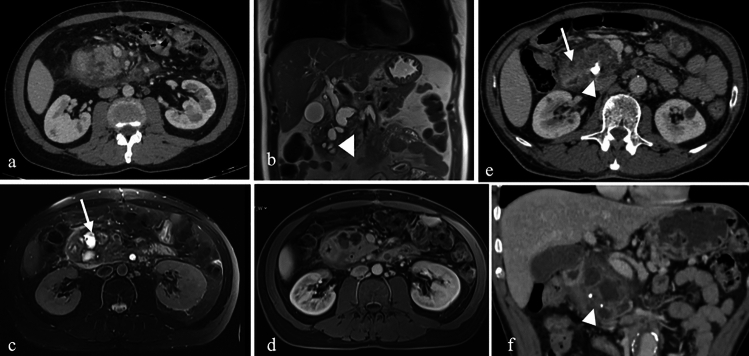
a. Axial IV contrast-enhanced CT, **b**. Coronal T2-weighted, **c**. Axial fat-saturated T2-weighted, **d**. Post-contrast fat-saturated T1-weighted MRI images of a 71-year-old male patient with paraduodenal pancreatitis. Fat stranding and focal fluid collections in the pancreaticoduodenal groove, as well as cystic changes in the duodenal wall, are visible (arrows). Additional findings include dilatation of the common bile duct, choledochus, and pancreatic duct (arrowhead). **e**. Axial and **f**. Coronal IV contrast-enhanced CT images of another patient (67-year-old female) with similar findings, along with calcifications within the cystic changes (arrowhead)

On CT, paraduodenal pancreatitis manifests as fat stranding in the pancreaticoduodenal groove and cystic changes in the second (D2) segment of the duodenum. Advanced cases may demonstrate delayed contrast enhancement within the groove and dilation of the common bile duct. MRI findings include a sheet-like mass between the pancreatic head and the duodenum, with low signal intensity on T1-weighted images and variable signal intensity on T2-weighted images, depending on the chronicity of inflammation. Post-contrast MRI typically demonstrates delayed enhancement, similar to CT [21] (Fig. 9).

#### Duodenal perforation

Duodenal perforation can result from inflammatory, traumatic, or iatrogenic causes, and timely diagnosis is critical to enable prompt surgical intervention. Perforations secondary to inflammatory conditions, such as Crohn’s disease, gallbladder inflammation, peptic ulcers, or NSAID overuse, are more commonly observed in the first (D1) segment of the duodenum. In contrast, traumatic and iatrogenic perforations more frequently involve the second (D2) segment [22].

Traumatic duodenal injuries account for 3%–5% of abdominal trauma cases and are often associated with injuries to adjacent organs. Penetrating trauma is the most frequent cause, particularly affecting the second (D2) segment [23]. Iatrogenic injuries are commonly linked to ERCP, esophagogastroduodenoscopy, stent placement, and sphincterotomy, with perforation and hemorrhage being the most significant complications [24].

**Fig. 10:**
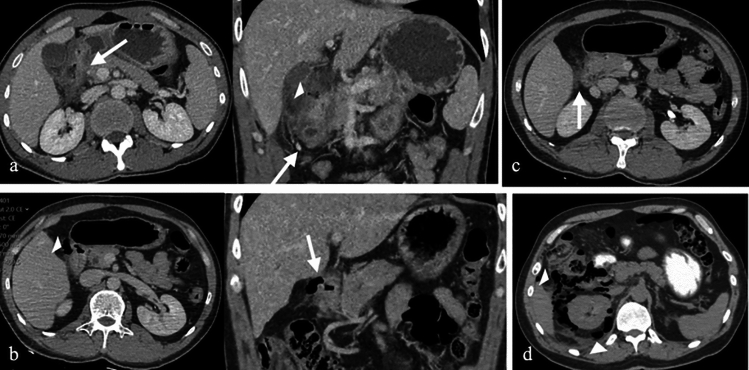
CT images from four different patients with duodenal perforation: **a**. Axial and **b**. Coronal IV contrast-enhanced CT images of a 34-year-old female and a 44-year-old male patient with duodenal ulcer perforations, showing diffuse duodenal wall thickening, periduodenal fat stranding, and free intra-abdominal air (arrows and arrowheads). **c**. Axial IV contrast-enhanced CT image of a 23-year-old female patient with post-traumatic duodenal perforation. **d**. Axial IV contrast-enhanced CT image of a 37-year-old male patient with duodenal perforation due to ERCP complications, demonstrating extensive retroperitoneal and periduodenal free air densities (arrowheads)

CT is the gold standard for diagnosing duodenal perforation, allowing for the identification of focal wall defects, extraluminal free air, and associated findings such as periduodenal fat stranding, edema, or hematoma [25] (Fig. 10).

#### Bouveret’s syndrome

Bouveret’s syndrome is a rare cause of gastric outlet obstruction, occurring when a large gallstone passes through a bilioduodenal fistula into the duodenal bulb. Gallstones larger than 2–2.5 cm in diameter are typically implicated, and the syndrome predominantly affects elderly patients with multiple comorbidities [26].

**Fig. 11:**
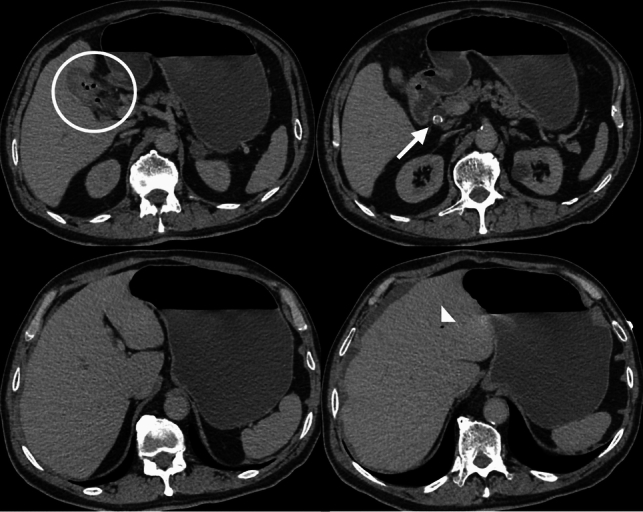
IV contrast-enhanced axial CT images of a 63-year-old female patient with Bouveret’s syndrome. A bilio-duodenal fistula with gallbladder wall thickening and air densities is noted (circle). A large gallstone is impacted in the duodenal bulb (arrow). Gastric dilatation and pneumobilia are evident, consistent with Rigler’s triad

On CT imaging, the classic diagnostic triad—known as Rigler’s triad—includes pneumobilia, gastric dilatation, and an ectopic gallstone within the gastrointestinal tract [26] (Fig. 11).

#### Duodenal hematoma

Duodenal intramural hematomas are rare and may result from blunt trauma, coagulopathy, iatrogenic interventions, severe peptic ulcer disease, or pancreatitis [27].

**Fig. 12:**
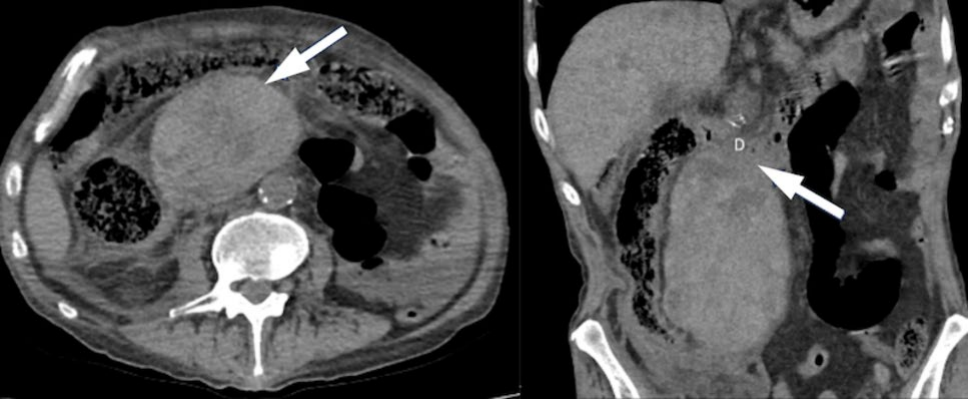
Non-enhanced CT images of an 87-year-old female patient, demonstrating an intramural duodenal hematoma affecting the second (D2) and third (D3) segments of the duodenum (arrows)

CT imaging is instrumental in diagnosis, revealing a high-density mass within the duodenal wall corresponding to a subacute hematoma. Late-stage findings, such as increased lesion density (70–90 HU), indicate clotted blood (Fig. 12). Contrast-enhanced CT can further localize active bleeding sites. MRI typically shows a hyperintense lesion on T1-weighted images, consistent with the hematoma’s blood content [27].

### Neoplastic pathologies

#### Duodenal lipoma

Duodenal lipomas are benign mesenchymal tumors composed predominantly of adipose tissue. They represent the third most common tumor type in the duodenum. These lesions are typically slow-growing and asymptomatic, often discovered incidentally during imaging or endoscopy. However, larger lipomas may cause obstructive symptoms, bleeding, or jaundice [28].

**Fig. 13:**
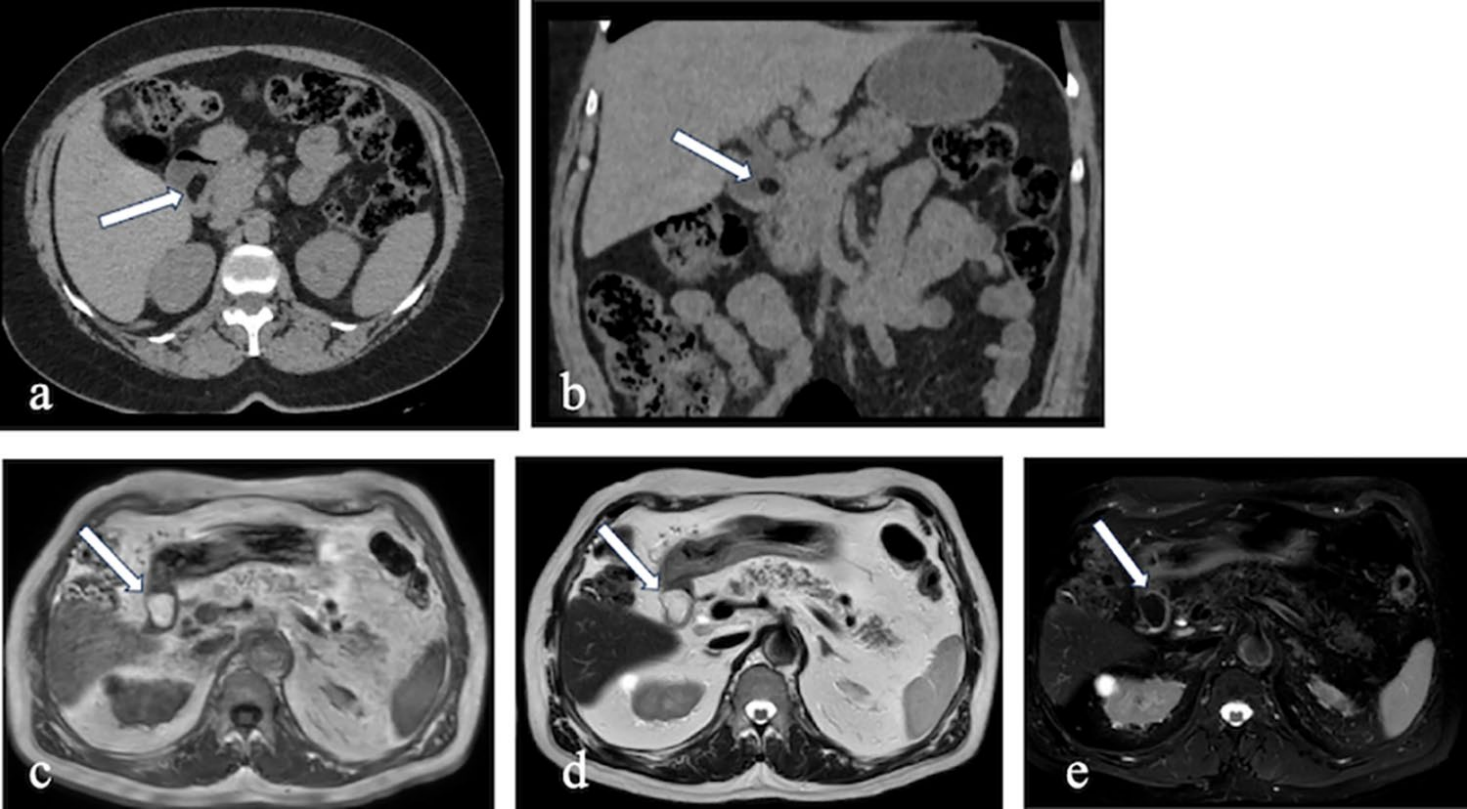
a. Axial and **b**. Coronal non-enhanced CT images of a 37-year-old male patient with a duodenal lipoma, exhibiting fat attenuation. MRI images **c**. T1-weighted, **d**. T2-weighted, and **e**. fat-saturated T2-weighted of another patient (58-year-old female) showing a lesion with high signal intensity on T1- and T2-weighted images, with signal suppression on fat-saturated sequences (arrows)

CT is a reliable imaging modality for diagnosing duodenal lipomas, which appear as well-defined lesions with fat density (−60 to −120 HU). MRI findings include high signal intensity on T1- and T2-weighted images, with signal suppression on fat-saturated sequences (Fig. 13). Larger lipomas may exhibit calcification, necrosis, or cystic degeneration, which can complicate diagnosis [28].

#### Duodenal polyp

**Fig. 14:**
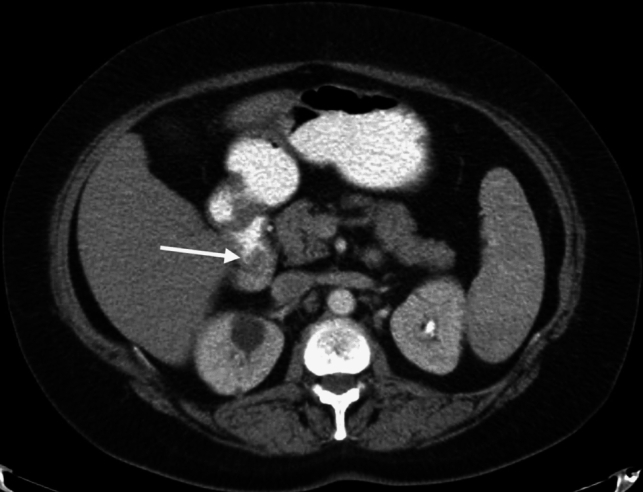
Axial IV contrast-enhanced CT image of a 39-year-old female patient showing a well-defined, isodense filling defect (arrow), diagnosed as an epithelial polyp

Duodenal polyps, particularly those smaller than 2 cm, are usually benign and solitary. Among epithelial polyps, adenomatous polyps are the most common and are subclassified into tubular, tubulovillous, and villous types [29] (Fig. 14).

Imaging findings include well-defined soft tissue lesions, either sessile or pedunculated, with mild and homogeneous contrast enhancement [30].

#### Leiomyomas

Leiomyomas are benign mesenchymal tumors of smooth muscle origin. Duodenal leiomyomas are relatively rare but may present as solitary submucosal masses causing gastrointestinal bleeding or, in rare cases, intussusception when they grow larger [31].

**Fig. 15:**
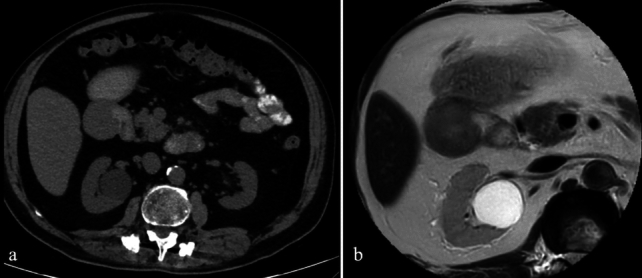
a. Non-enhanced axial CT image, **b**. Axial T2-weighted MRI image of a 62-year-old male patient, showing a round, well-defined, homogeneous lesion diagnosed as leiomyoma

On CT, leiomyomas appear as round or oval, well-defined solid masses. Larger lesions may exhibit calcification or ulceration. MRI findings typically demonstrate homogeneous signal intensity with moderate contrast enhancement. Differentiating leiomyomas from other submucosal tumors, such as schwannomas or gastrointestinal stromal tumors (GISTs), can be challenging, especially when calcification or ulceration is present [32] (Fig. 15).

#### Duodenal gastrointestinal stromal tumors (GISTs)

GISTs are the most common mesenchymal tumors of the digestive tract, primarily originating from the stomach or small intestine. Duodenal GISTs are relatively rare, accounting for less than 5% of all GIST cases, with the majority arising from the second (D2) segment of the duodenum [33].

Tumors smaller than 5 cm with fewer than five mitoses per high-power field are generally considered benign, whereas larger lesions with adjacent organ invasion, peritoneal seeding, or liver metastasis are classified as malignant. Approximately 10% of malignant duodenal tumors are GISTs [1].

**Fig. 16:**
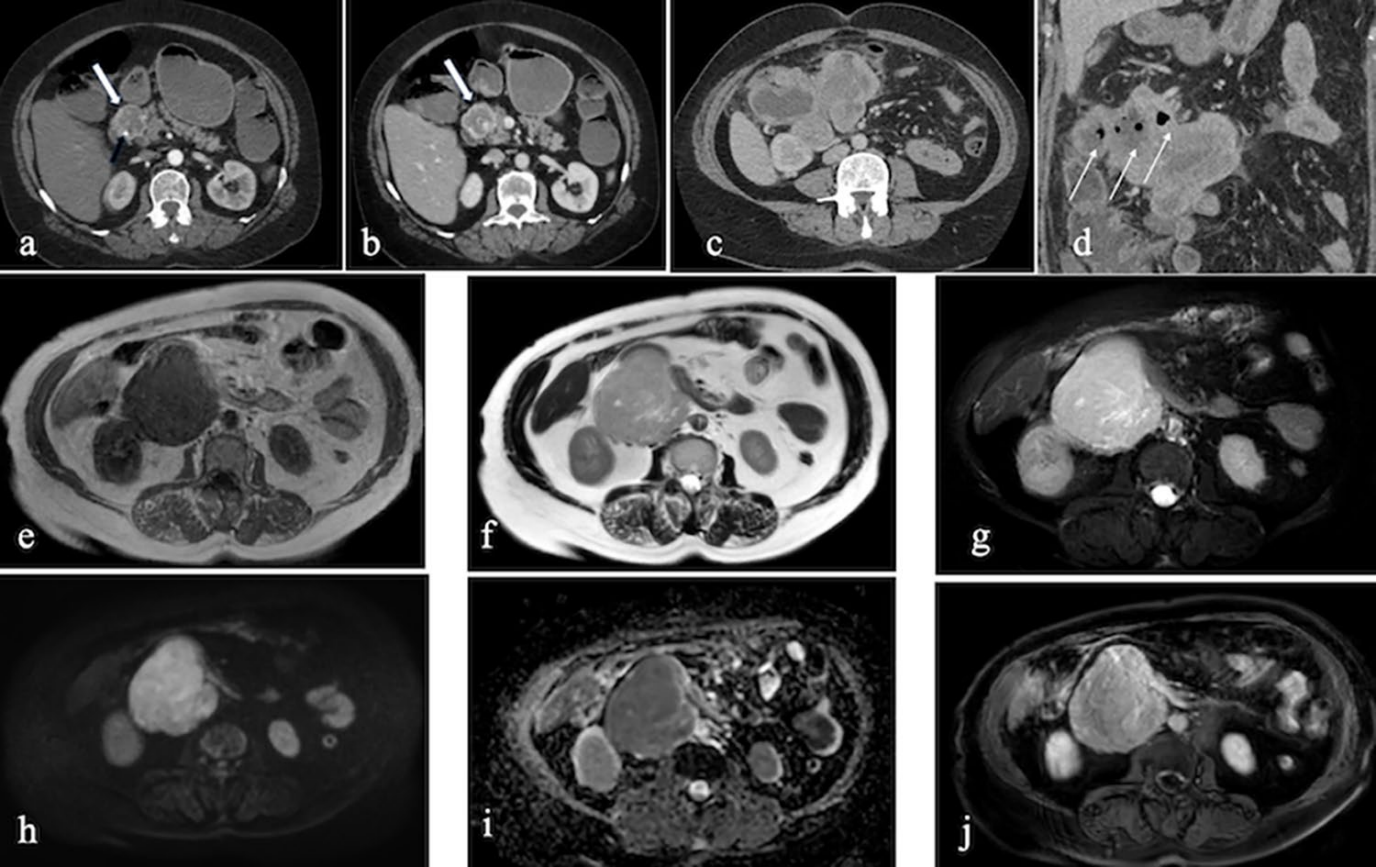
Axial IV contrast-enhanced CT images of two patients with duodenal GISTs: **a**, **b** A 50-year-old male patient with a well-demarcated hypervascular lesion containing focal calcifications (thick arrow). IV contrast-enhanced **c**. Axial, **d**. Coronal CT images of a 67-year-old male patient with a larger lesion containing air densities, indicative of necrosis (thin arrow). **e**. Axial T1-weighted, **b**. Axial T2-weighted, **c**. Axial fat saturated T2-weighted, **d**. Diffusion weighted, **e**. ADC maps and **f**. Contrast enhanced fat saturated T1-weighted MRI images of a 58-year-old female patient diagnosed with GIST, demonstrating hypervascularity, diffusion restriction, and heterogeneous enhancement

Clinically, GISTs may present with abdominal discomfort, gastrointestinal bleeding, or anemia. On CT, GISTs appear as well-demarcated, hypervascular lesions with homogeneous contrast enhancement. Larger tumors may exhibit necrosis, cystic changes, or central calcifications. MRI findings typically include low signal intensity on T1-weighted images and high signal intensity on T2-weighted images, though necrosis or hemorrhage may alter these characteristics [33] (Fig. 16).

#### Neuroendocrine tumors (NETs)

Neuroendocrine tumors (NETs) account for 2%–8% of all gastrointestinal tumors, with the majority occurring in the first (D1) and second (D2) segments of the duodenum. Gastrinomas are the most common type of duodenal NET, comprising 65% of cases, with approximately one-third being functional. Somatostatinomas are the second most common and are frequently located near the Ampulla of Vater [21].

Duodenal carcinoids, another subtype of NETs, rarely present with classic carcinoid syndrome and are often nonfunctional. Common symptoms include abdominal pain, bowel obstruction, and gastrointestinal bleeding. Periampullary carcinoids may also cause jaundice due to bile duct obstruction [21].

**Fig. 17:**
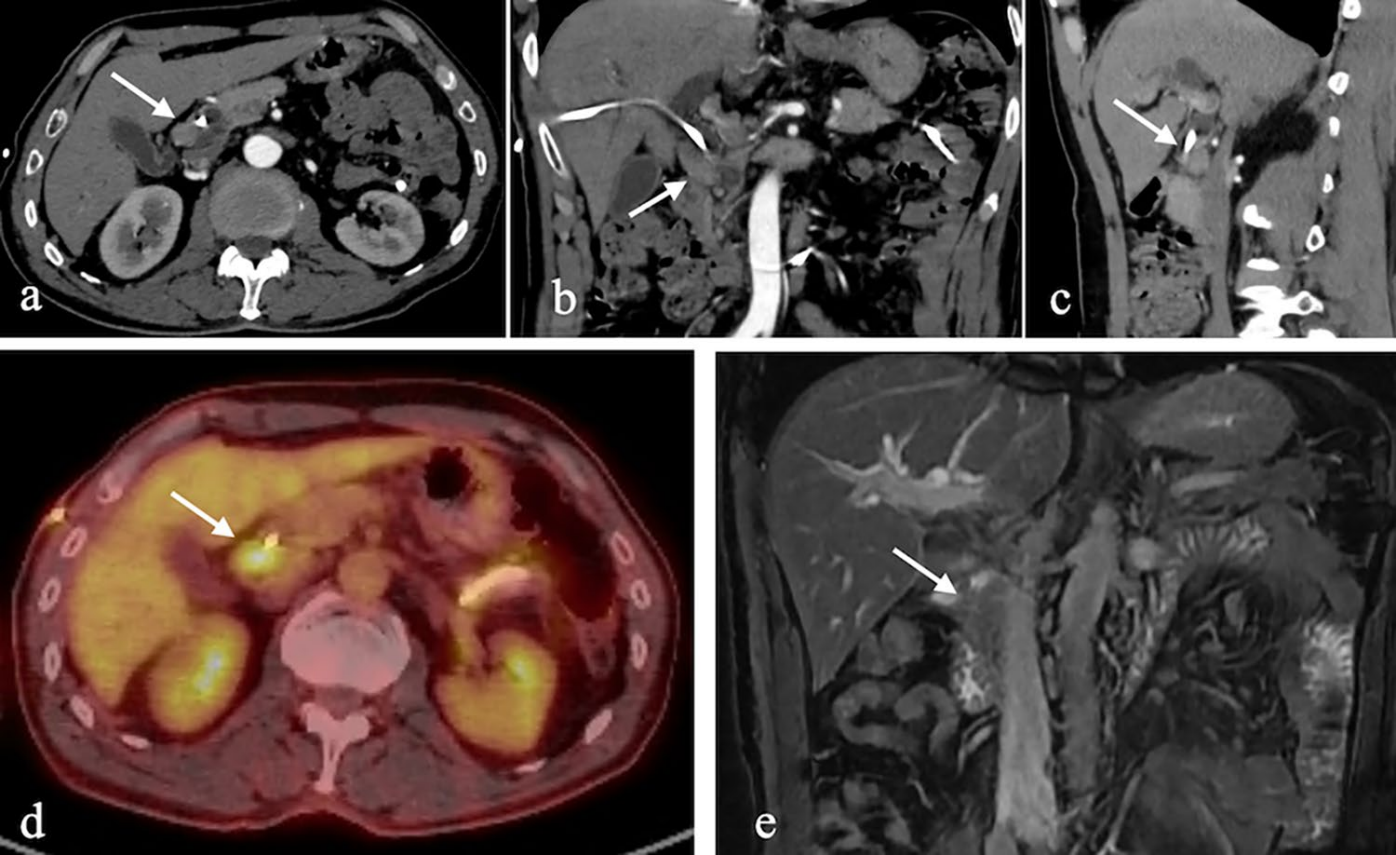
a. Axial, **b**. Coronal and **c**. Sagittal IV contrast-enhanced arterial-phase CT images of a 63-year-old male patient, showing a hypervascular nodular lesion in the second (D2) segment near the major papilla. **d**. FDG-PET/CT fusion image showing high FDG uptake. **e**. Coronal T2-weighted MRI image demonstrating hypointensity of the lesion. Diagnosis: neuroendocrine tumor

On CT, NETs may appear as polypoid lesions, intramural masses, or areas of wall thickening, often exhibiting early arterial-phase enhancement. MRI may reveal hyperintense cystic components on T2-weighted images. The presence of a periduodenal desmoplastic reaction and enhancing lymph nodes, even without an identifiable primary lesion, strongly suggests NETs. Periampullary NETs tend to have aggressive behavior and may present with distant metastases, most commonly to the liver [21] (Fig. 17).

#### Duodenal adenocarcinoma

Duodenal adenocarcinoma is the most common primary malignant tumor of the small intestine, accounting for 80%–90% of all primary duodenal malignancies [34].

Clinically, symptoms are nonspecific and may include abdominal pain, nausea, vomiting, jaundice, occult gastrointestinal bleeding, obstruction, and unintentional weight loss [34].

**Fig. 18:**
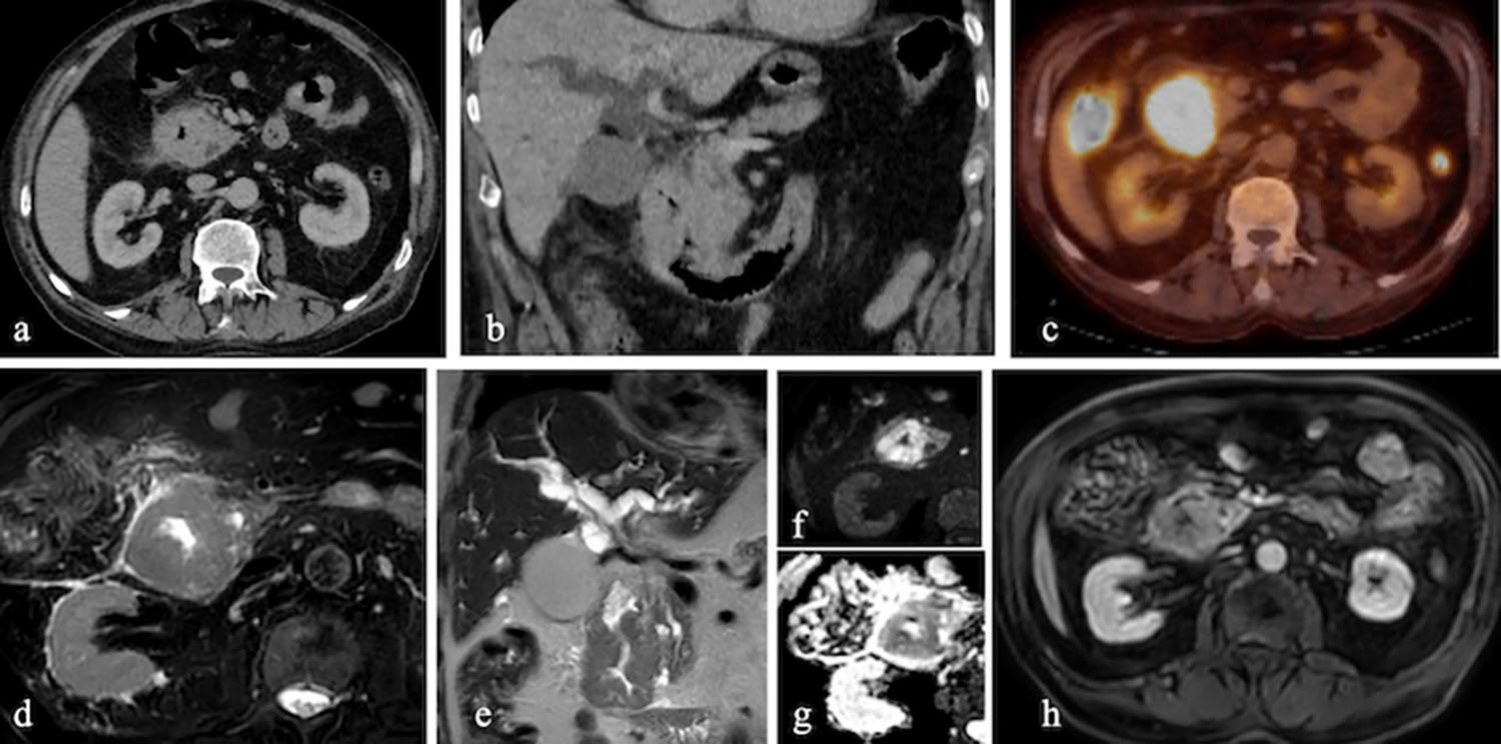
IV contrast enhanced **a**. Axial, **b**. Coronal CT, **c**. FDG-PET CT fusion images and **d**. Axial T2-weighted **e.** Coronal T2-weighted **f**. Diffusion weighted, **g**. ADC map and **h**. IV contrast enhanced fat saturated T1-weighted MR images of a 53-year-old female patient with duodenal mass diagnosed as adenocarcinoma. There is concentric wall thickening with heterogeneous enhancement. There is prominent FDG uptake. Lesion has low signal in T2-weighted imaging and shows diffusion restriction. As a result of choledochal involvement, there is dilatation in intrahepatic biller system

On CT, adenocarcinomas appear as asymmetric or concentric wall thickenings or mass lesions with polypoid growth patterns. Features such as necrosis, ulceration, and delayed heterogeneous contrast enhancement are also observed. MRI findings include low T2-weighted signal intensity relative to intraluminal contents and diffusion restriction, which improves diagnostic sensitivity and specificity. Peripheral lymph node involvement, locoregional invasion, and distant metastases, particularly to the liver, are common. The "double-duct sign," indicative of bile and pancreatic duct dilation, is frequently observed in periampullary adenocarcinomas [21] (Fig. 18).

#### Duodenal lymphoma

Duodenal lymphomas may occur as primary or secondary malignancies. Most cases involve T-cell or non-Hodgkin lymphomas, with approximately 30% exhibiting aggressive behavior, while the remainder follow an indolent course [35].

**Fig. 19:**
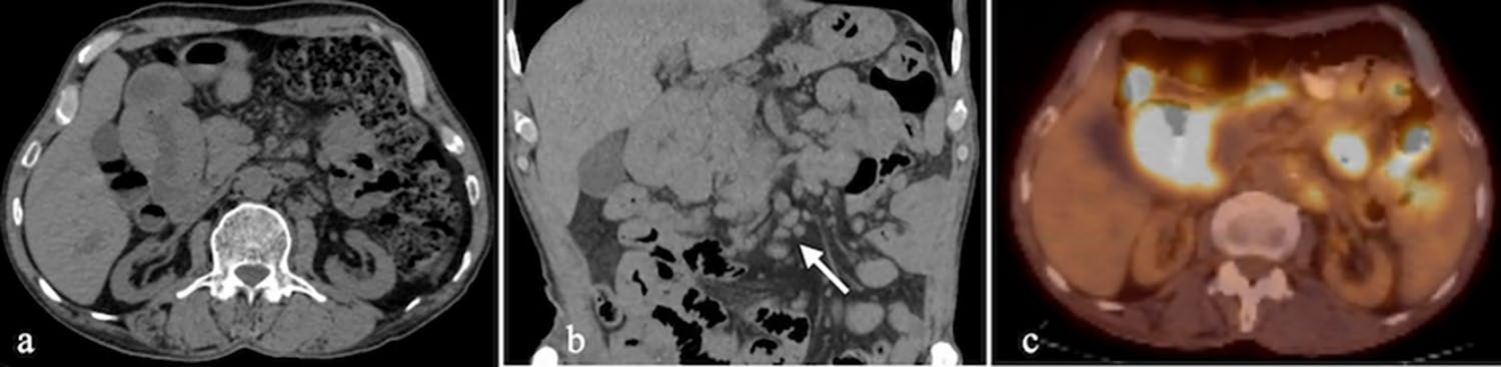
Non-enhanced CT **a**. Axial, **b**. Coronal, **c**. FDG-PET CT fusion images of a 22-year-old male patient diagnosed as T-cell lymphoma. There is a diffuse segmental wall thickening and prominent FDG uptake compatible with lymphoma involvement of the second—(D2) segment of duodenum. Additionally, there are spheric mesenteric lymph nodes (Arrow)

Radiologically, duodenal lymphomas often manifest as segmental wall thickening or large eccentric masses with potential necrosis and cavitation. Aneurysmal dilatation of the duodenum due to circumferential wall thickening is a hallmark feature [31]. Accompanying findings may include splenomegaly, mesenteric lymphadenopathy, or the "sandwich sign," characterized by lymph nodes flanking mesenteric vessels [35] (Fig. 19).

#### Metastases

Duodenal metastases are uncommon and may result from direct invasion, hematogenous spread, or peritoneal seeding. Primary malignancies that commonly metastasize to the duodenum include lung cancer, renal cell carcinoma, melanoma, and breast cancer, often via hematogenous dissemination. Direct invasion may occur from adjacent organs such as the pancreas, stomach, colon, and liver. Additionally, mucinous tumors of the ovary, appendix, and colon can spread to the duodenum through peritoneal seeding [21].

**Fig. 20:**
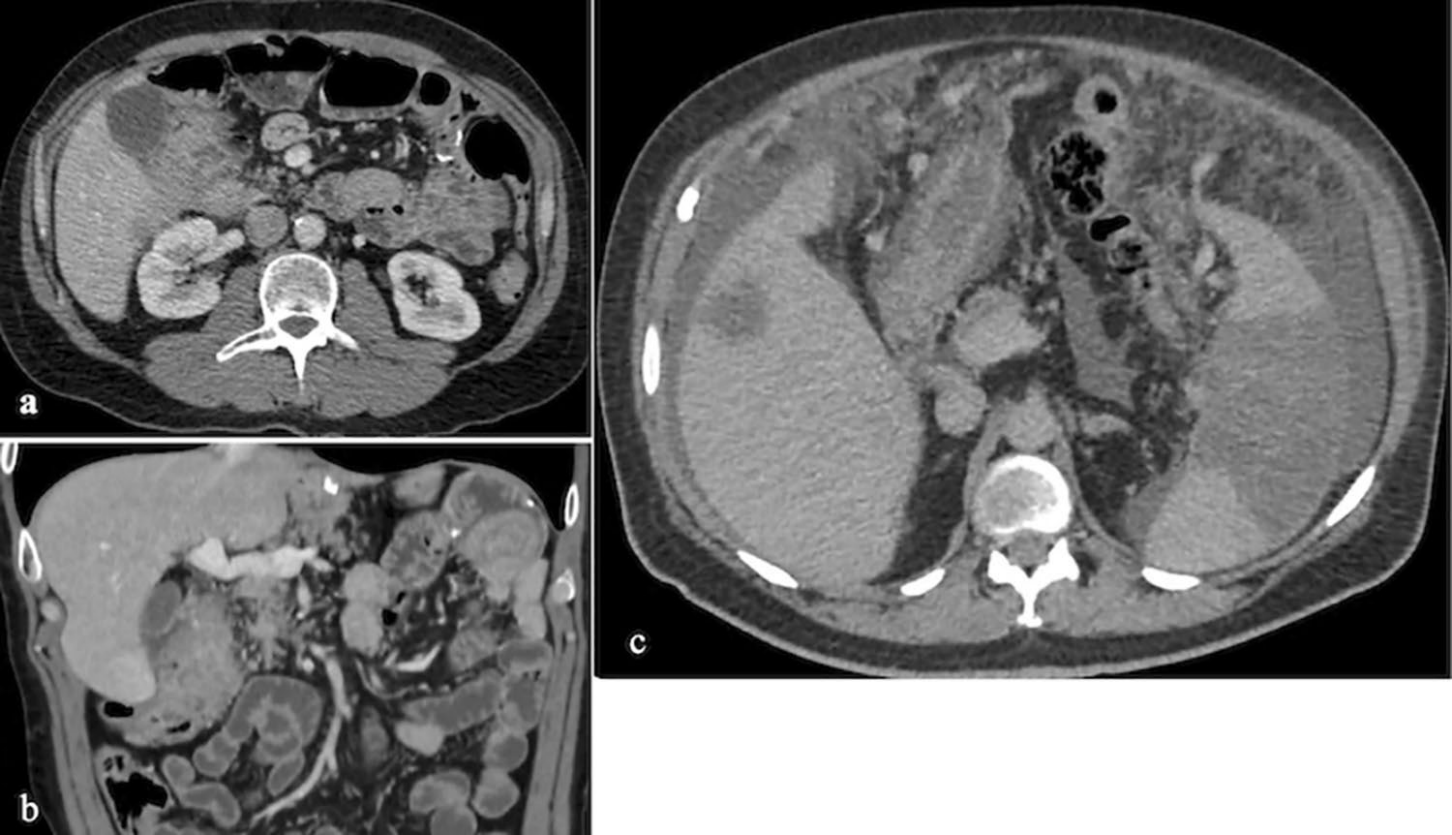
IV contrast enhanced **a**. Axial and **b**. Coronal CT images of a 48-year-old male patient with duodenal metastasis of gastric tumor located at cardia. There is an irregular solid mass with necrotic changes and heterogenous enhancement. **c**. IV contrast enhanced axial CT image of a 52-year-old female patient with colon carcinoma and peritoneal metastasis. In the second—(D2) segment of duodenum there is irregular wall thickening, wall enhancement and paraduodenal fluid collection. Additionally, there are peritoneal nodular lesions, mental heterogeneity and metastatic mass at the fifth segment of liver

Imaging findings of duodenal metastases are nonspecific and may include well-defined lesions with homogeneous enhancement or irregular masses with adjacent tissue invasion and ulceration [21] (Fig. 20).

## Conclusion

Misdiagnosis or delayed diagnosis of pathologies in the second (D2) segment of the duodenum can lead to significant morbidity and mortality. Cross-sectional imaging modalities serve as the reference standard for evaluating these conditions, with CT being the primary imaging technique. Recognizing the typical imaging features, anatomical localizations, and clinical presentations of duodenal pathologies is essential for accurate diagnosis and differential diagnosis. Furthermore, familiarity with D2 segment pathologies helps reduce unnecessary examinations and minimizes misdiagnosis rates.

## Data Availability

No datasets were generated or analysed during the current study.
